# Pandemic hospitals and reorganizing emergency departments

**DOI:** 10.3906/sag-2106-169

**Published:** 2021-12-17

**Authors:** Afsin Emre KAYIPMAZ, Ahmet DEMİRCAN

**Affiliations:** 1 Department of Emergency, Ankara City Hospital, Ankara Turkey; 2 Member of COVID-19 Scientific Advisory Board of Ministry of Health; 3 Department of Emergency, Faculty of Medicine, Gazi University, Ankara Turkey

**Keywords:** COVID-19 pandemics, disasters, emergency treatment, emergencies

## Abstract

Emergency departments have always been the first point of contact for hospitals in many situations, including man-made and natural disasters. The first places where patients with symptoms of COVID-19 were met in health institutions were also emergency departments. Emergency departments play an important role in diagnosing the disease and isolating patients (by hospitalization if necessary). The process, which starts with the triage of outpatients admitted to the emergency department and brought by ambulance, continues as isolation of the patients in appropriate areas including physical evaluation, management of laboratory and scanning processes and, if necessary, providing cardiopulmonary resuscitation with airway support. Afterwards, patients can be treated as an outpatient, or hospitalized, or treated at the intensive care unit in line with their preliminary diagnosis and clinical conditions. Disruptions that may occur in one or more of these stages can lead to crowds and lengthy queues in the emergency department by prolonging the follow-up period of the patients. One of the strengths of Turkey at this point is that emergency departments are accustomed to the heavy patient load. The experiences gained from these conditions have facilitated the organization of pre-hospital emergency medical services, pandemic hospitals, and their emergency departments. In this organization, the main goal should be to provide uninterrupted and high-quality patient care through personnel training, personal protection measures, and the creation of physical conditions. Turkey’s emergency departments are accustomed to managing the intensive patient flow, as they work at full capacity during normal times. Thanks to the experiences of emergency healthcare workers, health service was provided without any patient being turned away from the door of the emergency departments during the COVID-19 pandemic. In this review, we aimed to present the organization of pandemic hospitals and emergency departments during the COVID-19 pandemic. We made a schematic representation of the architectural areas through the emergency department of Ankara City Hospital, which has a bed capacity of 4200 with 256 beds in emergency department.

## 1. Introduction

The COVID-19 disease caused by the SARS-CoV-2 virus, which was first detected in China’s Hubei state Wuhan in December 2019, was categorized as a pandemic on March 11, 2020, by the World Health Organization [1]. Emergency departments have always been the first point of contact for hospitals in many situations, including man-made and natural disasters. The first places where patients with symptoms of COVID-19 were met in health institutions were also emergency departments. Emergency departments play an important role in diagnosing the disease and isolating patients (by hospitalization if necessary) [2].

The organization of emergency departments is of great importance in times of disaster, where a large number of patient applications are made in a short time. Hospitals and emergency departments must have an action plan against such situations. The patient flow can be maintained without any problems only with this plan.

The process, which starts with the triage of outpatients admitted to the emergency department and brought by ambulance, continues as isolation of the patients in appropriate areas, physical evaluation, management of laboratory and scanning processes and, if necessary, providing cardiopulmonary resuscitation with airway support. Afterwards, patients can be treated as an outpatient, or hospitalized, or treated at the intensive care unit in line with their preliminary diagnosis and clinical conditions. Disruptions that may occur in one or more of all these stages can lead to crowds and lengthy queues in the emergency department by prolonging the follow-up period of the patients. 

Taking into account the fact that the pandemic is a condition of disaster, reorganizing the pandemic hospitals and emergency departments is one of the steps that should be taken before the first cases appear in the country. As a matter of fact, the emergency departments in the Republic of Turkey started to preparations long before the pandemic was declared. One of the strengths of Turkey at this point is that emergency departments are accustomed to the heavy patient load. It has been reported that the number of annual applications to the emergency department is higher than Turkey’s population [3]. Due to the habit of people to use the emergency departments for simple and chronic health problems [4] 7 days/24 h, it was ensured that the emergency departments, which are accustomed to meet intensive patient applications, are also ready for the management of disaster situations such as pandemics. Although this is an inappropriate use, the experiences gained from these conditions have facilitated the organization of pre-hospital emergency medical services, pandemic hospitals, and their emergency departments. In this organization, the main goal should be to provide uninterrupted and high-quality patient care through personnel training, personal protection measures, and the creation of physical conditions [5].

In this review, we aimed to present the organization of pandemic hospitals and emergency departments during the COVID-19 pandemic. We made a schematic representation of the architectural areas through the emergency department of Ankara City Hospital, which has a bed capacity of 4200 with 256 beds in emergency department, and 696 beds in intensive care units.

### 1.1.Pandemic hospitals

The hospitals where the examination, treatment, and isolation processes of patients suspected or diagnosed with COVID-19 were to be carried out, have been determined as “Pandemic Hospitals” by the Ministry of Health of the Republic of Turkey. Pandemic hospitals were selected from among hospitals that provide tertiary intensive care services and where at least two major specialists from infectious diseases and clinical microbiology, internal medicine, and pulmonary diseases are present together or brought together by assignment. In conditions where pandemic hospitals are not available, health institutions with secondary intensive care beds could also provide services in the status of pandemic hospital [6,7]. 

The establishment of pandemic hospitals enabled segregation of hospitals where hematology-oncology, bone marrow transplant, radiation oncology patients, or solid organ transplant patients are followed and, in this way, it was ensured that COVID-19 patients and immunosuppressed patients are not treated in the same environment [7].

For this purpose, COVID-19 patients detected in the emergency departments of hospitals which were not pandemic hospitals were immediately transferred to pandemic hospitals. In the emergency departments of pandemic hospitals, for the purposes of prevention of transmission, it is necessary not to bring together COVID-19 patients and cardiovascular, neurological emergencies, trauma, and similar patient groups who applied to the emergency department for other reasons in waiting rooms and treatment observation areas [8].

Despite all precautions, positive cases can also be detected in emergency observation units where patients without COVID-19 are followed. For this reason, both the proper use of masks by all patients admitted to the emergency department and their attendants, and the proper use of personal protective equipment by healthcare professionals according to the area of work and the medical intervention are critical. In addition, in order to prevent crowd and in-hospital contamination, it is important to apply to primary health care institutions before applying to the emergency departments of pandemic hospitals for simple health problems other than COVID-19. Postponing nonemergency medical interventions and surgical procedures during periods when the number of patients increases and limiting daily polyclinic examinations to appointments only and to certain time intervals also allow the prevention of crowding and the provision of higher priority health services to urgent and critical patients [9]. Here, the point to be considered in the hospital is to prevent the patients who have applied to the hospital without an appointment and whose condition is not urgent, from creating a crowd there by being admitted to the emergency department. Otherwise, the crowd that nonemergency patients and their relatives will create in the waiting areas of the emergency departments may bring these areas into risk in terms of contagion.

The establishment of “Hospital Pandemic Boards” and an emergency crisis coordination desk directly connected to the management have been one of the extremely useful practices in order to ensure inter-clinical coordination in the management of the pandemic in hospitals. In these committees, situations related to emergency departments, COVID-19 outpatient clinics, inpatient services, and intensive care units are discussed in detail and the opportunity to make decisions with a common mind is created. Hospitals can make decisions in these committees to increase or decrease the capacity allocated to COVID-19 in line with the pandemic plans and according to the current number of cases.

## 2. Reorganization of physical spaces

### 2.1. Triage and COVID-19 polyclinics

**Figure 1:**
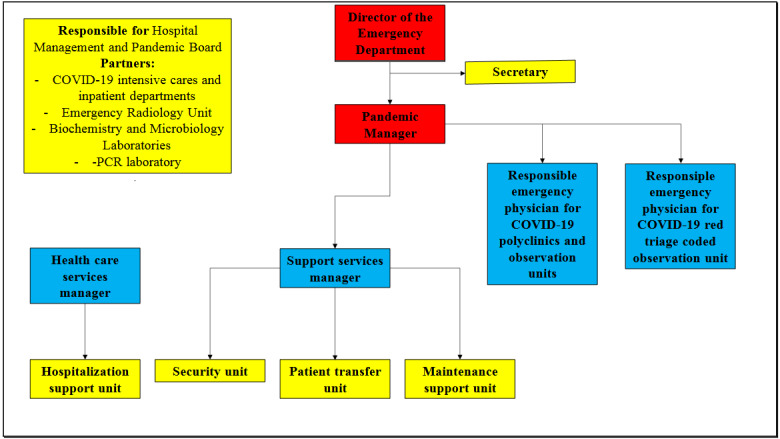
Responsibility plan in emergency departments of pandemic hospitals.

Since the emergency departments are the only areas where hospitals can apply at any time of the day without the need for an appointment, these departments are also very crowded areas apart from pandemics and disasters. This crowd increases especially during nonworking hours. It is stated that a great majority of patients will apply to the emergency departments during the pandemic process, and it is stated that there is a need for preparation accordingly [10]. We showed the flow of health services in the emergency department in Figure 1.

As with all epidemics that spread rapidly in the world, it was important for hospitals to be prepared for this situation with the increase in the number of cases for COVID-19 as well. For this reason, hospitals started their preparations months before the first case was declared in Turkey. The areas where the cases would be admitted were determined, and the ways to be followed in diagnosis and treatment were defined. 

The training of emergency healthcare workers, who would take care of the cases, was also at the forefront of the preparations made. In the meetings organized by the education coordinators of the hospitals, general information about the disease was shared with the employees, as well as the rules that must be followed from the use of personal protective equipment and hand hygiene were reminded in detail. Flow charts were prepared for patient management and hung in the appropriate places of the emergency departments. These charts included the use of personal protective equipment, the care to be taken in the approach to the patient, the steps to be followed in the management of the airway in the red areas, and in the management of cases with cardiopulmonary arrest. In the clinic, faculty members closely followed the developments related to diseases from the world literature. 

To guide the field regarding the COVID-19 disease, about which there was not much information at the onset, Coronavirus Scientific Advisory Board of the Republic of Turkey Ministry of Health’s has prepared guidelines. In these guidelines, there is a door triage inquiry guide for outpatients [11].

The guide includes questions that healthcare professionals wearing appropriate personal protective equipment will ask outpatients about possible COVID-19 symptoms. It is evaluated whether the patients who enter through the door are suspected of COVID-19 with three basic questions: 

1) Whether there is a fever or a history of fever.

2) Whether there is a cough.

3) Presence of symptoms such as sore throat, headache, shortness of breath, muscle aches, loss of taste and smell, diarrhea.

The patient showing any of these symptoms is asked to wear a mask and directed to the predetermined area. In addition, epidemiological history is also questioned in the guideline. Especially it is questioned whether any of the relatives of the patient has been diagnosed with COVID-19 or whether they have applied to the hospital due to respiratory disease in the last 14 days. The history of travelling abroad was also one of the questions sought to be answered in the early stages of the epidemic. 

The most appropriate option is to divide the triage areas for outpatients in the emergency department into two as preliminary triage and emergency triage. Pre-triage can be indoors in hospitals with sufficient physical facilities, and it can be in places such as tents and containers, which are formed in front of emergency departments in hospitals with limited space. Patients who arrive at the pre-triage area should be evaluated according to the above-mentioned guideline; if it is a possible positive case, they are directed to the area reserved for COVID-19. If it is not a possible positive case, they are directed to the triage area in the emergency department. Patients arriving by ambulance are evaluated by the triage personnel at the ambulance entrance, a possible positive case is taken to the emergency and critical care areas designated for COVID-19 [9,10].

**Figure 2:**
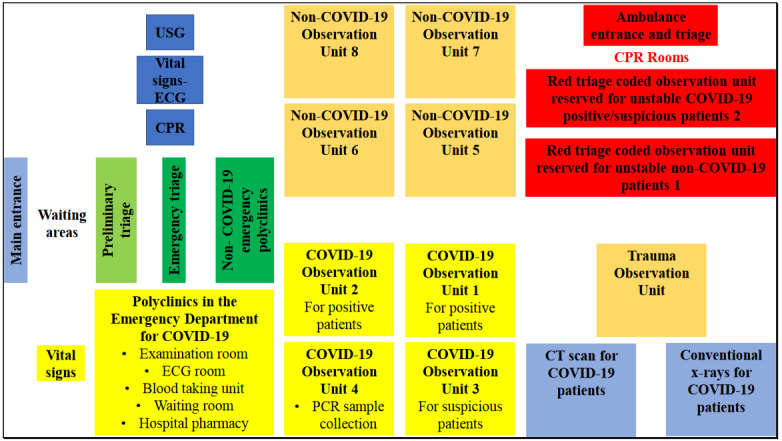
Diagram of COVID-19 and non-COVID-19 treatment observation areas in the reorganization of Ankara City Hospital Emergency Department. Each observation area has a capacity of 24 stretchers. Yellow and red observation areas reserved for COVID-19 patients have negative pressure. There is also an isolation room in each of the yellow areas. In addition, there are a total of 12 rooms in the red area, each of which is isolated and can accommodate two patients. Abbreviations: ECG: Electrocardiogram, CPR: Cardiopulmonary resuscitation, PCR: Polymerase chain reaction test, USG: Ultrasonography. Vital signs: Fever, pulse, blood pressure, respiratory rate, fingertip oxygen saturation.

In this review, we made a schematic representation of the architectural areas through the emergency department of Ankara City Hospital, which is the largest health institution in Turkey and Europe and accepted as the reference hospital in the COVID-19 pandemic. (Figure 2) Ankara City Hospital has a bed capacity of 4200 with 256 beds in emergency department, and 696 beds in intensive care units.

Thermal cameras or digital noncontact thermometers placed in pre-triage are useful applications in terms of detecting the body temperature of patients entering the emergency department. “Fever screening”, which was first used in the SARS epidemic in March 2003, has also been a method used in various countries around the world to distinguish high-risk people from other people [12].

At the entrance of the outpatient clinic where the patient with suspected/diagnosed COVID-19 is referred from the preliminary triage, vital signs including body temperature, pulse, blood pressure, respiratory rate, and fingertip oxygen saturation are checked. During the period when the number of patients is on the rise towards the peak, shift polyclinics can be put into use in the polyclinic areas of the hospital to prevent the concentration of polyclinics established in or near the emergency department. Possible cases with stable vital signs and simple complaints can be referred to these polyclinics, which will also provide service at intervals to be determined outside of working hours. In this way, the crowd in the outpatient clinics in the emergency department could be reduced. If the number of patients in the community tends to decrease and the outpatient clinics in the emergency department are able to handle the patient load, the examination and treatment processes of these patients could be resolved in these areas without referral to other polyclinics. Waiting areas must be created in accordance with the physical distance rules for patients who can wait for outpatient examination results. Considering that the results of the polymerase chain reaction (PCR) test may take hours, patients who are decided to be followed up and isolated may be sent home with isolation measures to wait for the test results, and if necessary, antiviral treatments are given to them from the hospital pharmacy. The point to be considered here is whether the discharged patient has isolation opportunities. In this regard, if patients coming from places where they live collectively, such as military units, prisons, dormitories, nursing homes, do not have isolation facilities in the institutions they come from, it should be ensured that isolation periods are spent in areas to be determined by the provincial health administrations. University dormitories, public guesthouses, or medical observation clinics of hospitals can be designated as suitable areas for this purpose. In addition, it should be kept in mind that in cases where many people live together in a large family-style in a small house without isolation facilities, a large number of new patients may return due to clusters caused by patients who cannot comply with isolation. These people should also be observed in the isolation areas to be determined, even if their condition is stable.

The use of public transport should be avoided as much as possible for patients who are diagnosed with COVID-19 and are planned to be discharged and do not have their private vehicles. For this purpose, it is important to establish an urban transportation system that will take positive patients who will be discharged from the emergency department to their places of residence.

### 2.2. COVID-19 observation areas with yellow triage code

The examinations and treatments of patients who are hospitalized in inpatient services in the emergency departments are carried out in special areas created for these patients. It is ensured that patients already diagnosed are monitored in the “positive patient observation area” and possible positive cases are followed up in the “suspicious patient observation area”. It is easier to create these areas and transform them into negative pressure areas in more modern hospitals with large physical space, such as the newly built Turkish City Hospitals, than in old buildings. In old buildings, it is useful to determine areas where non-COVID-19 patients, COVID-19 patients, and separate patients will be followed to prevent cross-contamination. 

It is necessary to transfer the patients with a pre-diagnosis and an indication for hospitalization to the relevant services without waiting in the observation areas of the emergency department if there is availability in the inpatient services. Establishing a hospitalization support unit to determine the empty beds in the services, determining the appropriate empty beds, and ensuring the smooth transfer of the patients from the emergency department to these services are beneficial in terms of accelerating the patient flow in the emergency department. 

 It should be kept in mind that COVID-19 may be detected incidentally in patients presenting with asymptomatic or atypical symptoms in non-COVID-19 areas such as trauma, which are considered low risk for COVID-19 in the emergency department. In a study conducted in Turkey, it was reported that only 48% of incidentally detected positivity in trauma patients had a history of suspicious contact. For this reason, it is essential that healthcare personnel working in assumed low-risk areas pay attention to the use of personal protective equipment as well [13].

### 2.3. COVID-19 observation areas with red triage code

Red triage coded observation areas are reserved for the care of high-risk, unstable patients with a diagnosis or suspicion of COVID-19. Aerosol-generating procedures such as endotracheal intubation, noninvasive mechanical ventilation, manual ventilation, and tracheotomy, which have been shown to increase the risk of contamination, are frequently performed in these areas [14]. For this reason, it is imperative that healthcare professionals working in these areas use personal protective equipment such as FFP2/N95 masks, glasses, aprons, bonnets, and gloves [15].

Airway opening methods to be applied in red areas are aerosol-forming procedures. Oxygen support can be given to patients with a nasal cannula, a simple face mask, or a reservoir (nonrebreather) mask in both yellow and red coded COVID-19 observation areas. Among these methods, which are defined as conventional methods, the FiO2 values reached when oxygen is given at most 6 L/min by nasal cannula and 8 liters/min by simple face mask are at most 45% and 60%, respectively. FiO_2_ can rise above 85% when 10–15 liters/min of oxygen is given with a reservoir mask. If the oxygenation has not improved despite this, high flow oxygen application (HFNC) is one of the applications that can be done in the red area if there is a device. Increasing the current in HFNC to a maximum of 60 liters/min and keeping the FiO_2_ value below 60% will also protect the patient from the undesirable effects of oxygen. Noninvasive mechanical ventilation can also be applied for selected patients who do not need urgent intubation (continuous positive airway pressure/bilevel positive airway pressure). Although it is difficult to do on stretchers in the emergency department, the prone position can also be tried. In patients who need to be intubated, this procedure should be performed by the most experienced healthcare worker to shorten the time and increase the success of the first pass. In the meantime, a rapid sequence intubation protocol should be applied. The use of a video laryngoscope will benefit the operator. Viral and bacterial filters must also be used in circuits [16,17].

Patients stabilized in the red area should be transferred to the designated areas in the intensive care units without delay according to their PCR test positivity or their PCR negative but clinical/imaging compatibility. With the transfer of patients to intensive care units, emergency departments should be circulated and made ready to accept new patients. In the absence of available intensive care beds in the relevant hospital, the referral of patients to the nearest most suitable hospital should be provided under the supervision of the local health authority and through their ambulances. 

### 2.4. COVID-19 emergency radiology units

During the pandemic, the number of noncontrast thoracic tomography scans for three-dimensional imaging of the thorax in the emergency department has increased significantly. However, in the period between the end of February and mid-May of 2020, when the pandemic first peaked, it was determined that the emergency radiological examinations decreased by 46% compared to the normal time. It is thought that closure decisions, “stay at home” calls, postponement of elective procedures and the decrease in the number of emergency department applications had an impact on this [18].

It would be appropriate to determine separate devices for tomography and ultrasonography examinations of patients who are followed up with suspected COVID-19 in emergency departments. Since ultrasound is an examination performed with the patient and the operator standing at a close distance, usually less than 1 m, the operator must perform the procedure using personal protective equipment suitable for the job he/she is doing. Technicians and the personnel who clean after the patient should also pay attention to personal protection during the taking and removal of the patient to the tomography devices. It is important that the patient also wears a mask [19,20].

For hospitals that do not have the opportunity to allocate separate devices for patients with suspected COVID-19, cleaning, and disinfection of devices after the examination of each patient, are carried out following the principles of “Infection Control in terms of COVID-19 in radiology units” in the Infection Control and Isolation Guide. For cleaning purposes, 1/100 diluted bleach (Cas No: 7681-52-9) or chlorine tablets are used. If the surfaces have encountered the patient’s body fluids (vomit), these substances are diluted 1/10 and used for cleaning these surfaces. Disinfectants containing at least 70% alcohol can also be used for the same purpose [19,20].

### 2.5. Emergency laboratory services

Biochemistry laboratories work to assist in the diagnosis of COVID-19 and to detect the levels of variables that show a poor prognosis such as lymphocyte count, C-reactive protein, d-dimer, and ferritin. Microbiology laboratories are used for tests such as blood culture, urine culture, and stool sample examination, which are requested from the emergency department to detect the focus of infection.

According to the data of the General Directorate of Public Health of the Ministry of Health, as of May 30, 2021, there are 480 COVID-19 diagnostic laboratories authorized by the General Directorate of Public Health throughout the country. PCR tests are carried out in these laboratories. These laboratories in the main health service hospitals of the provinces, university hospitals, and private hospitals enable the tests taken within the same hospital to be studied quickly. 

It would be helpful if the areas from which PCR test samples were taken for COVID-19 were turned to negative pressure, if possible. The PCR sample can be taken by a trained healthcare professional. It is essential for the healthcare worker to wear the appropriate personal protective equipment, especially masks in N95 or FFP2 class [21].

The samples taken should be delivered to the PCR laboratories, where the tests will be carried out, after the registration process is completed. Reducing the process of transporting and delivering the tests taken in the emergency department to the laboratory will speed up the test results. It is useful for the laboratory team to know that the tests of these patients are prioritized by printing the barcodes of the samples to be taken from the red triage coded observation areas where critical patients are observed, on a different colored paper and delivered to the laboratory. 

## 3. The effect of the pandemic on emergency department practices

In the first months when the cases were on the rise in Turkey, it was determined that the “nonurgent” practices, which are called green triage code applications, decreased statistically significantly compared to the same period of the previous year. This situation arises since people do not prefer to come to the emergency department for bearable, simple symptoms due to the fear of being infected in hospitals [22].

It was found that while the number of COVID-19 patients increased in the first 8 weeks of the epidemic in Italy, non-COVID-19 applications decreased dramatically. It was also found that trauma patients significantly decreased. It has been speculated that this is largely related to the absence of traffic and the cessation of sports activities due to quarantine. In addition, it was emphasized that the decrease in the applications of patients with serious complaints such as chest pain and abdominal pain should be considered [23].

Similarly, in the United States, while the number of emergency department applications due to COVID-19-related disease increased in the early stages of the pandemic, the total number of emergency department applications decreased significantly. In addition, it was concluded that the awareness of the society should be increased for urgent situations such as acute myocardial infarction, stroke, and more deadly than COVID-19 [24]. In Turkey, information has been given through media organs so that patients with complaints that may cause more serious problems, especially if the diagnosis is delayed, should not hesitate to apply to the hospital. 

In Italy, which is one of the European countries where the devastating effects of the epidemic are observed most, re-organization of the emergency departments has become necessary with the increasing number of cases. Within this context, all the waiting areas of the emergency departments have been transformed into emergency rooms, each containing oxygen and electricity supply. Since all elective procedures were stopped, physicians and nurses from surgical and internal specialties who were available were assigned to the emergency department and they were provided to work in accordance with predetermined protocols. Italian physicians state that the most important lesson they have learned is that hospitals should be proactive and prepared in terms of health workers, medical equipment, putting new beds into operation, and organizing intensive care beds flexibly [23].

Physicians from developed countries such as the USA, Italy, and Spain define this extraordinary situation as a “perfect storm” in common language. In the first months of the epidemic, scientists emphasized that if preventive measures to prevent human-to-human transmission are not taken, this may result in a tragedy. It was emphasized that fighting the disease by joining forces with science and medicine is the only way to win this war [25]. 

## 4. Conclusion

In cases of a pandemic, as in all disasters, emergency departments are the first clinics that patients are admitted to. Therefore, emergency departments should always be ready for such extraordinary situations. In this sense, it is important to provide continuous training to personnel on disasters and epidemics. For communicable diseases, hospitals and emergency departments should have predetermined the areas where patients will be isolated. Arrangements should be made so that capacity can be increased if necessary. 

Turkey’s emergency departments are accustomed to managing the intensive patient flow, as they work at full capacity during normal times. Thanks to these experiences of emergency healthcare workers, health service was provided without any patient being turned away from the door of the emergency departments during the COVID-19 pandemic. The previous experiences, the preparations made before the first case was announced in Turkey, and the patient flow in the moments when the number of cases reached the peak play an important role in this. 

In the future we may encounter pandemics like COVID-19 that can easily be transmitted from person to person through the respiratory tract. Thanks to the experience gained from COVID-19, the emergency departments in Turkey will be able to manage similar pandemic situations with the right reorganization.
